# A transcriptome-based risk model in sepsis enables prognostic prediction and drug repositioning

**DOI:** 10.1016/j.isci.2024.111277

**Published:** 2024-10-28

**Authors:** Qiuyue Long, Hongli Ye, Shixu Song, Jiwei Li, Jing Wu, Jingsong Mao, Ran Li, Ke Li, Zhancheng Gao, Yali Zheng

**Affiliations:** 1 Department of Respiratory, Critical Care and Sleep Medicine, Xiang’an Hospital of Xiamen University, School of Medicine, Xiamen University, Xiamen 361101, China; 2 Institute of Chest and Lung Diseases, Xiamen University, Xiamen 361101, China; 3 Department of Critical Care Medicine, Xiang’an Hospital of Xiamen University, School of Medicine, Xiamen University, Xiamen 361101, China; 4 Department of Respiratory and Critical Care Medicine, Peking University People’s Hospital, Beijing 100044, China; 5 Department of Vascular Intervention, Guilin Medical College Affiliated Hospital, Guilin Medical College, Guilin 541000, China

**Keywords:** Health sciences, Intensive care medicine, Internal medicine, Medical microbiology, Medical specialty, Medicine, Pharmacology

## Abstract

Septic management presented a tremendous challenge due to heterogeneous host responses. We aimed to develop a risk model for early septic stratification based on transcriptomic signature. Here, we combined genes OLAH, LY96, HPGD, and ABLIM1 into a prognostic risk score model, which demonstrated exceptional performance in septic diagnosis (AUC = 0.99–1.00) and prognosis (AUC = 0.61–0.70), outperforming that of Mars and SRS endotypes. Also, the model unveiled immunosuppressive status in high-risk patients and a poor response to hydrocortisone in low-risk individuals. Single-cell transcriptome analysis further elucidated expression patterns and effects of the four genes across immune cell types, illustrating integrated host responses reflected by this model. Upon distinct transcriptional profiles of risk subgroups, we identified fenretinide and meloxicam as therapeutic agents, which significantly improved survival in septic mice models. Our study introduced a risk model that optimized risk stratification and drug repurposing of sepsis, thereby offering a comprehensive management approach.

## Highlights

• A risk model for septic prognosis was construed by LY96, HPGD, OLAH, and ABLIM1
• The high-risk subgroup was associated with an immunosuppressive profile
• The low-risk subgroup had a poor response to hydrocortisone
• The model screened fenretinide and meloxicam for septic treatment

## Graphical abstract

**Figure:**
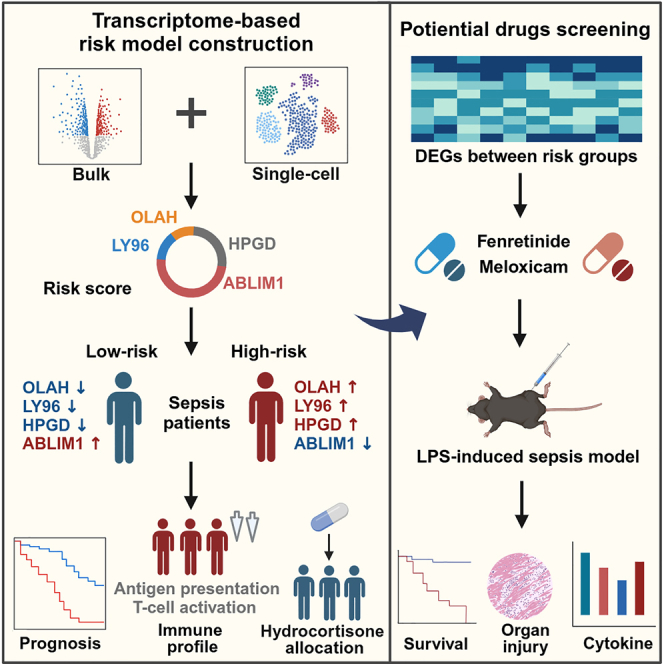

## Introduction

Sepsis is a life-threatening organ dysfunction caused by a dysregulated host response to infection.^1^ Patients with sepsis accounted for 16–36.4% of all hospitalization deaths,^2^ with a high pre-discharge mortality rate of 41.9% in the intensive care unit (ICU).^3^ Despite the introduction of the surviving sepsis campaign in 2004^4^ and extensive research efforts, the mortality rate remains high, underscoring the persistent challenge sepsis poses to healthcare systems. The deficiency in early sepsis recognition and the varied host responses to standard treatments contribute to the suboptimal outcomes in sepsis management. This underscores the necessity for innovative approaches to patient stratification and tailor treatments to their unique conditions.

Sepsis exhibits substantial genetic heterogeneity due to the diverse responses of thousands of genes to infection.^5^ Transcriptomic profiling has enabled the classification of sepsis into distinct molecular types, such as Mars (molecular diagnosis and risk stratification of sepsis)^6^ and SRS (sepsis response signatures)^7^^,^^8^ system. The Mars system delineates sepsis into four distinct molecular endotypes, with Mars1 being associated with the highest mortality, characterized by the BPGM and TAP2 genes. In contrast, other endotypes like Mars3 have shown a better prognosis and are linked to an intact adaptive immune response. Similarly, the SRS system identifies immunosuppressive status SRS1, which is linked to favorable responses to steroid therapy but is also associated with higher early mortality.^7^^,^^8^ These molecular endotypes reflect sepsis patients’ varied inflammatory responses, immune states, and drug reactions, highlighting the potential for personalized therapeutic approaches. Identifying these subtypes is a step toward developing targeted therapies that could improve clinical outcomes for high-risk patients by facilitating early and precise intervention.

Transcriptomic profiling is a crucial tool in the quest for precision medicine, as it captures the integrated effects of pathogen-host and therapy-host interactions. A gene signature reflecting a specific host’s response is essential for guiding therapy. For example, the transcriptomic framework SELECT has been instrumental in stratifying cancer patients and predicting patient responsiveness to targeted therapies and immunotherapies, thereby aiding in precise medical decisions in oncology.^9^ Furthermore, transcriptomes have been utilized to screen for potential drugs for conditions, such as obesity, non-alcoholic steatohepatitis, and hyperuricemia, demonstrating their utility in identifying effective treatments.^10^ Numerous molecules have been investigated for septic therapy, such as Capsaicin and Celastrol reported by Wang and his colleagues.^11^^,^^12^ However, despite these advances, current endotype studies in sepsis, such as Mars and SRS, have not been matched with specific molecular targets or therapeutic agents. The benefits of these systems in developing tailored treatments for sepsis remain largely undiscovered. Therefore, we aimed to identify patients at high mortality risk and select targeted therapies based on their immune molecular profiles, moving us closer to the goal of targeted therapies that improve clinical outcomes through early and precise intervention.

In the current study, we constructed a four-gene risk score to aid septic patients’ early diagnosis and prognosis. Our prognostic prediction capability was better than that of Mars and SRS. High-risk patients exhibited immunosuppression signatures, while low-risk individuals responded poorly to hydrocortisone. Drugs chosen according to the transcriptome feature showed encouraging therapeutic benefits in mice septic models. Our findings provided a more accurate predictive model and underscored the potential of tailored therapeutic strategies informed by individual immune profiles in improving sepsis treatment.

## Results

### Identification of prognostic genes and development of a risk score

**Figure 1:**
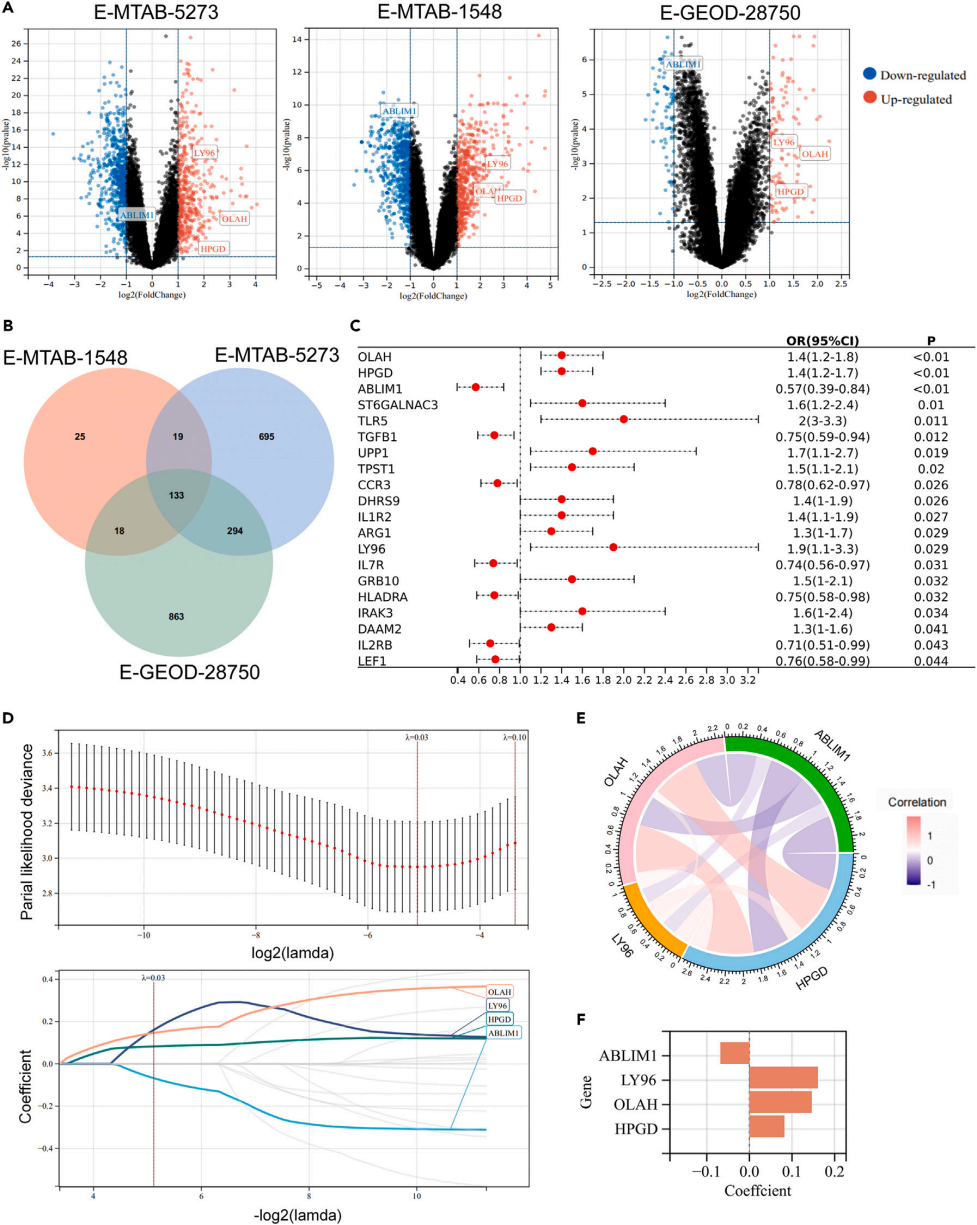
Construction of a risk model based on prognostic genes (A) Identification of differentially expressed genes (DEGs) in control and septic patient groups within the E-MTAB-5273, E-MTAB-1548, and E-GEOD-28750 datasets (FDR <0.05). (B) The 133 common DEGs shared across three derived datasets. (C) Univariate Logistic regression analysis yielded 20 prognostic genes (*p* < 0.05). (D) LASSO regression was conducted to identify signature genes between sepsis survivals and non-survivals. (E) Correlation network linking ABLIM1, LY96, OLAH, and HPGD. (F) The coefficients of the four key genes to calculate risk scores.

Firstly, differentially expressed genes (DEGs) between healthy and sepsis groups across three independent datasets were identified, yielding 1,308 DEGs in E-MTAB-1548, 1,141 DEGs in the E-MTAB-5273 dataset, and 195 DEGs in E-GEOD-28750 (Figures S1A–S1C and Figure 1A), respectively. Then, 133 shared DEGs (Figure 1B) were subjected to univariate logistic regression to assess their association with 28-day mortalities. A total of 20 predictive genes were identified, including 13 genes OLAH, HPGD, ST6GALNAC3, TLR5, UPP1, TPST1, DHRS9, IL1R2, ARG1, LY96, GRB10, IRAK3, and DAAM2 as risk factors of death outcomes (OR > 1, *p* < 0.05), and seven genes ABLIM1, TGFB1, CCR3, IL7R, HLADRA, IL2RB, and LEF1 as protective factors (OR < 1, *p* < 0.05) (Figure 1C and Table S3).

The LASSO regression analysis was then employed to refine the selection of genes for a predictive risk model. The determination of optimal lambda and regression coefficient of each gene were illustrated in Figure 1D. The probability of deviance was minimized when lambda (λ) reached a minimum value of 0.03. Based on this λ value, the number of included genes and their coefficients were determined. As shown in the lower panel in Figure 1D, the best performance of the model was reached when four genes, OLAH, LY96, HPGD, and ABLIM1, were included. The correlation network of these signature genes was shown in Figure 1E. The protective gene ABLIM1 expression was negatively correlated with the other three risk factors. Septic patients with lower expression of OLAH (HR = 1.57, 95%CI = 0.73–3.41), LY96 (HR = 2.13, 95%CI = 1.00–4.54), HPGD (HR = 2.41, 95%CI = 1.14–5.06) had a more favorable prognosis compared to those with higher levels, whereas higher expression of ABLIM1 was associated with better outcomes (HR = 0.39, 95%CI = 0.18–0.85), as shown in Figure S2A. The risk score was calculated using the following formula: risk score = −0.0678 ∗ ABLIM1 + 0.0825 ∗ HPGD +0.1607 ∗ LY96 + 0.1463 ∗ OLAH, with the coefficients for these genes detailed in Figure 1F.

### Validation of the risk score’s predictive and diagnostic utility

**Figure 2:**
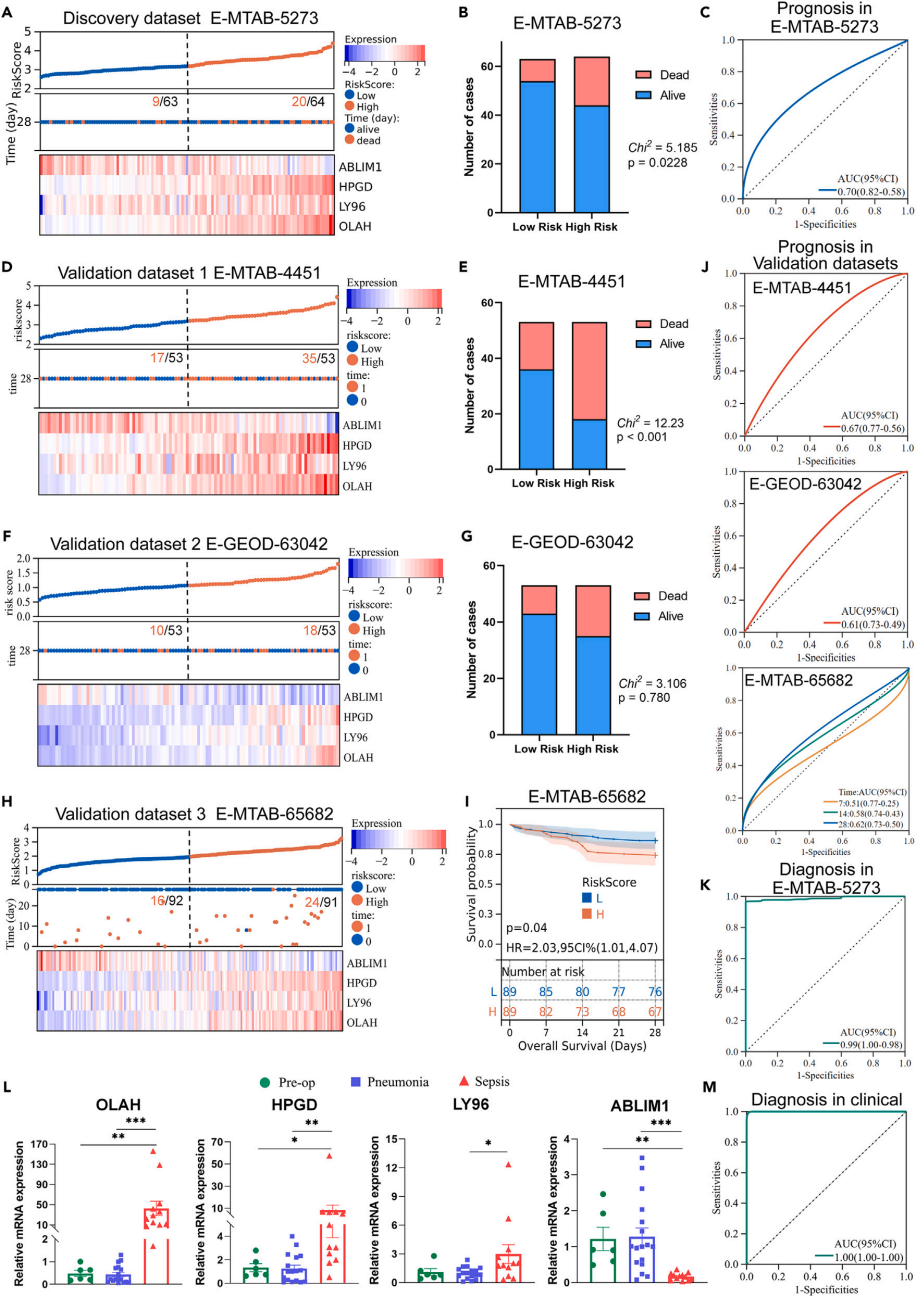
Internal and external validations of the risk model (A) The risk survival status chart of low-risk (LR) and high-risk (HR) subgroups in E-MTAB-5273, divided by the median value of the risk score. Low: Low risk, High: High risk. (B) The distribution of 28-day survival status in the HR and LR subgroups in E-MTAB-5273. (C) The ROC curve of the risk score for sepsis prognosis in E-MTAB-5273. (D) Risk survival status chart of the LR and HR groups in the validation dataset E-MTAB-4451, divided by the median value of risk score. Low: Low risk. High: High risk. 1: Death. 0: survival. (E) The distribution of 28-day survival status in HR and LR subgroups in E-MTAB-4451. (F) Risk survival status chart of LR and HR subgroups in the validation dataset E-GEOD-63042, divided by the median value of risk scores. (G) The distribution of 28-day survival status in HR and LR subgroups in E-GEOD-63042. (H) Risk survival status chart of LR and HR subgroups in the validation dataset E-MTAB-65682, divided by the median cutoff value of risk scores. (I) The Kaplan-Meier curve of risk subgroups in E-MTAB-65682. (J) ROC curves of the risk score for sepsis prognosis in three validation datasets. (K) ROC curves of the signature for sepsis diagnosis in the discovery dataset E-MTAB-5273. (L) RT-PCR results of the prognostic genes OLAH, LY96, HPGD, and ABLIM1 in clinical samples, including pre-operation (*n* = 6), pneumonia (*n* = 17), and sepsis patients (*n* = 12), as calculated by one-way ANOVA with nonparametric test. Results were represented as means with error bars representing the standard error of the mean (SEM). ∗*p* < 0.05, ∗∗*p* < 0.01, ∗∗∗*p* < 0.001. (M) The ROC curve of the risk score for sepsis diagnosis in the clinical samples.

We verified the predictive capabilities in both derivation and external validation datasets. In the discovery dataset E-MTAB-5273, sepsis patients were categorized into high-risk (HR) and low-risk (LR) subgroups based on the risk score’s median value. A lower expression level of ABLIM1 and higher expression levels of OLAH, HPGD, and LY96 were observed in HR patients, which correlated with increased mortality (Figures 2A and 2B). The area under curves (AUC) of the risk score was 0.70 (95%CI 0.58–0.82) for 28-day mortality prediction (Figure 2C), indicating its potential as a predictive tool.

Lower expression of ABLIM1 and higher expression of HPGD, LY96, and OLAH were also observed in external datasets—E-MTAB-4451, E-GEOD-63042, and E-MTAB-65682 (Figure 2D, 2F, and 2H). Further validation across the validation datasets consistently demonstrated a higher mortality rate in the HR group. Compared to the LR group, more decease cases were observed in the HR group in E-MTAB-4451 (35/53 vs. 17/53, *Chi**^2^* = 12.23, *p* < 0.01) and E-GEOD-63042 (10/53 vs. 18/53, *Chi*^2^ = 3.106, *p* = 0.780) (Figure 2E and 2G). The Kaplan-Meier analysis of the E-MTAB-65682 dataset showed significantly lower 28-day survival rates in the HR group (Figure 2I). The AUC for predicting 28-day mortalities were 0.67 (0.56–0.77) in E-MTAB-4451, 0.61 (0.49–0.73) in E-GEOD-63042, and 0.62 (0.50–0.73) in E-MTAB-65682, suggesting the risk score’s reliability in predicting patient outcomes (Figure 2J).

Moreover, we explored the risk score’s utility for early sepsis detection. Internal validation produced an AUC of 0.99 (0.98–1.00) for sepsis diagnosis (Figure 2K). Furthermore, the relative expression levels of the four prognostic genes were measured by RT-PCR in clinical samples. The results showed significantly higher levels of OLAH, HPGD, and LY96 in sepsis patients compared to those with preoperative conditions or pneumonia, increased by 95.16-, 6.58-, and 2.89-fold, respectively, while ABLIM1 was downregulated by 7.33-fold (Figure 2L). The risk score’s receiver operating characteristic (ROC) curve demonstrated excellent diagnostic sensitivity and specificity in clinical cases (sepsis vs. preoperative and pneumonia), with an AUC of 1.00, reinforcing its viability for early sepsis identification (Figure 2M).

### Comparative efficacy of the risk score with established sepsis endotypes

**Figure 3:**
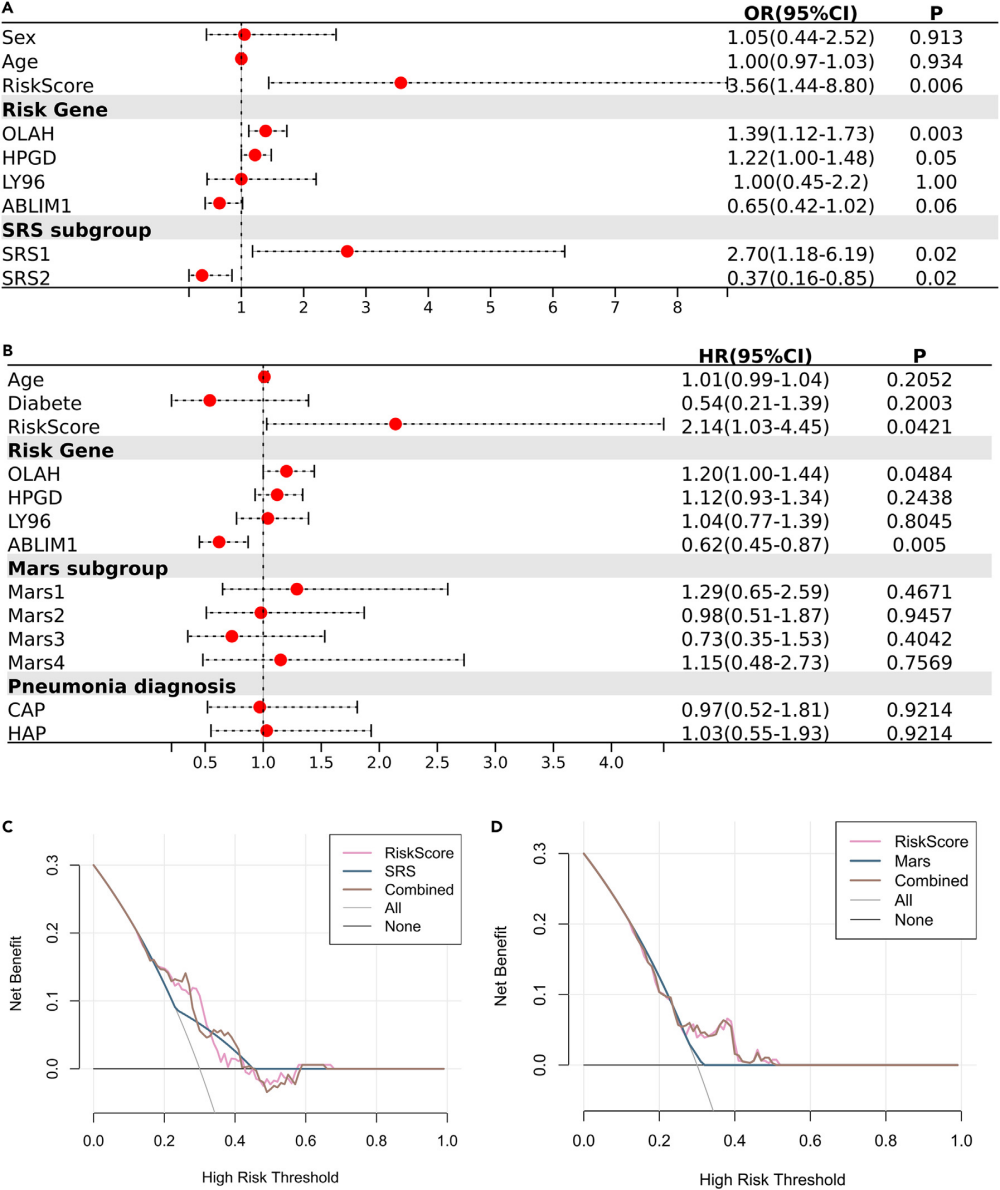
The risk score outperformed SRS and Mars in predicting 28-day mortality (A) Univariate Logistic regression analysis performed on the E-MTAB-4451 dataset, the original dataset of the SRS endotype. (B) Univariate Cox regression analysis performed on the E-MTAB-65682 dataset, the original dataset of the Mars endotype. (C and D) Comparative assessment of the risk model’s clinical utility against the SRS endotypes (C) and the Mars endotype (D) via decision curve analysis (DCA).

We conducted univariate regression analysis to evaluate the influence of various clinical factors and genetic endotypes on sepsis prognosis (Figure 3A and 3B). The analysis included demographic details, such as age, gender, and diabetes. Furthermore, we reassessed the predictive value of the risk score against well-established sepsis endotypes within their original datasets: the SRS endotype in E-MTAB-4451 and the Mars endotype in E-MTAB-65682.^6^^,^^7^ The demographic factors were unrelated to the sepsis prognosis. In E-MTAB-4451, the SRS1 endotype (OR = 2.70, 95%CI = 1.18–6.19) was a significant risk factor for 28-day mortalities, while the SRS2 endotype (OR = 0.37, 95%CI = 0.16–0.85) showed protective effects. In contrast, the Mars endotypes did not significantly affect clinical outcomes. Notably, the risk score, which integrates four prognostic genes, proved to be a more potent predictive indicator than individual genes like OLAH or the SRS1 endotype (HR > 1, *p* < 0.05), with higher odds ratios reflecting its stronger correlation with mortality in both datasets. Decision curve analysis (DCA) further demonstrated the risk score’s superior predictive accuracy over the SRS and Mars endotypes. The analysis showed more excellent net benefits for the risk score across a range of mortality thresholds, whether used alone or combined with existing endotypes (Figure 3C and 3D). These findings underscore the clinical relevance and potential of the risk score as a prognostic tool in sepsis management.

### Functional analysis revealed immunosuppression states in high-risk sepsis patients

**Figure 4:**
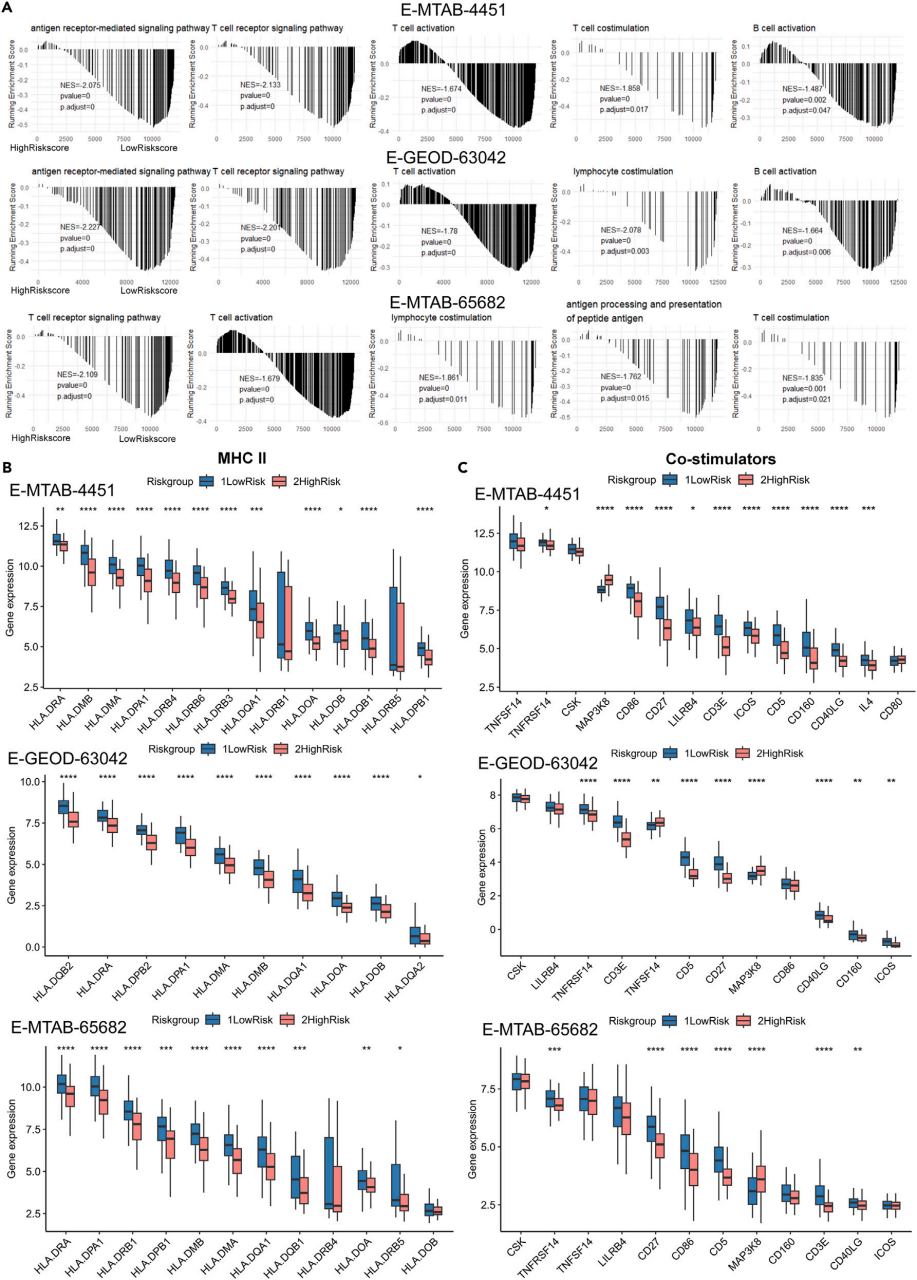
Functional explorations of risk subgroups revealed immunosuppressive status in the HR patients (A) Biological processes associated with antigen presentation and lymphocyte activation significantly enriched in the LR group rather than in the HR group, as estimated by GSEA. (B and C) Comparisons of gene expressions of (B) MHCII molecules or (C) co-stimulators between the HR and LR groups in E-MTAB-4451, E-GEOD-63042, and E-MTAB-65682, respectively. Data were represented as median with interquartile range. Statistical significance was calculated using the Wilcoxon test. ∗*p* < 0.05, ∗∗*p* < 0.01, ∗∗∗*p* < 0.001, ∗∗∗∗*p* < 0.0001.

Compared to the LR subgroup, the functional analysis of the HR subgroup in our study has shed light on the prevalent immunosuppressive states in HR individuals. By integrating three validation datasets, we performed Gene Ontology (GO) and Kyoto Encyclopedia of Genes and Genomes (KEGG) enrichment analyses, which identified critical cellular processes such as T cell activation and inflammatory response, indicative of immune-related mechanistic disparities between the risk subgroups (Figures S3A–S3C and S3D). Further investigation through gene set enrichment analysis (GSEA) revealed that biological processes central to adaptive immunity, including antigen receptor signaling, T cell co-stimulation, and activation of T and B cells, were predominantly enriched in the LR group (Figure 4A). This suggests a deficiency in the HR group’s capacity to initiate adaptive immune responses. Consistent with these findings, the expression of MHC-II molecules and co-stimulatory factors was significantly reduced in the HR group compared to the LR group (Figures 4B and 4C, *p* < 0.05).

Spearman correlation analysis underscored the immunosuppressive nature of the HR group, with positive correlations between ABLIM1 expression and the expression of MHC-II molecules (HLA-DRA, HLA-DPA, HLA-DQA, HLA-DOA, HLA-DOB, HLA-DMA, and HLA-DMB) and co-stimulatory factors (CD160, CD3E, CD27, CD40LG, CD5, CD86, DPP4, and ICOS), and negative correlations between the risk genes OLAH, LY96, and HPGD and the same immune factors (Figures S3E and S3F). These findings indicated that the risk score, at a transcriptional level, mirrored the dynamics of antigen presentation and T cell activation in sepsis patients, with the observed decreases in the HR group contributing to their immunosuppressive phenotype.

### Septic patients in the low-risk group responded poorly to hydrocortisone

**Figure 5:**
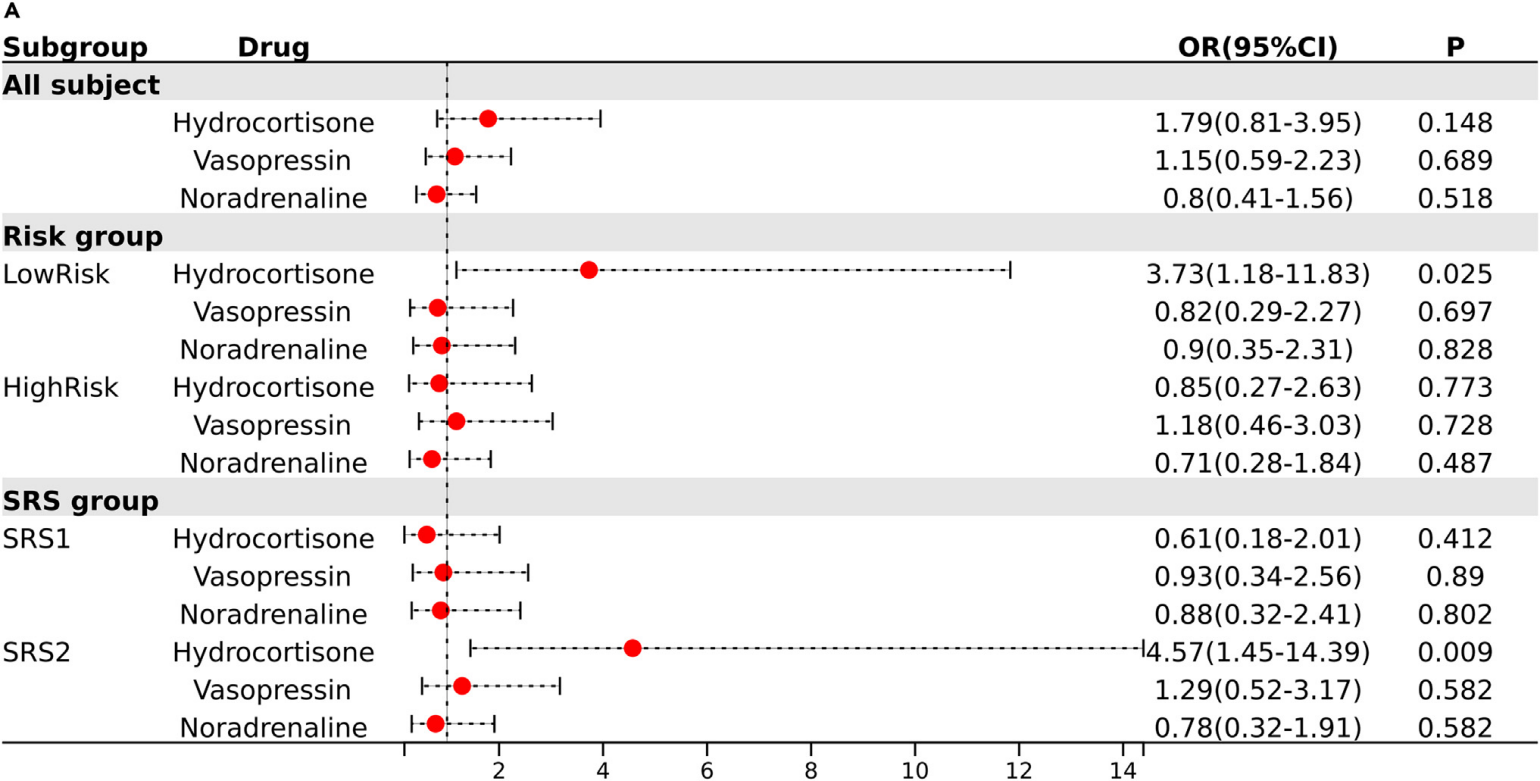
The LR subgroup responded poorly to hydrocortisone (A) The Logistic regression models integrated interactions between drug allocation and SRS or risk subgroups in the dataset E-MTAB-7581.

The E-MTAB-7581 dataset from the VANISH trial provided a unique perspective on the treatment outcomes of septic patients, who were categorized by their SRS endotypes and randomized to receive either vasopressin or norepinephrine, followed by hydrocortisone or placebo.^8^ The SRS2 endotype responded poorly to the use of hydrocortisone, as reported. However, our analysis of this dataset revealed that LR patients, characterized by active antigen presentation and T cell activation, unexpectedly exhibited a higher risk of adverse outcomes when treated with hydrocortisone (OR: 3.73, 95% CI: 1.18–11.83, *p* < 0.05) (Figure 5A). In contrast, there was no significant change in the prognosis of the HR subgroup, which was characterized by immunosuppression. By identifying patients more likely to respond poorly to hydrocortisone, our risk score could improve treatment strategies and patient outcomes in sepsis management.

### Drug repurposing for sepsis treatment

**Figure 6:**
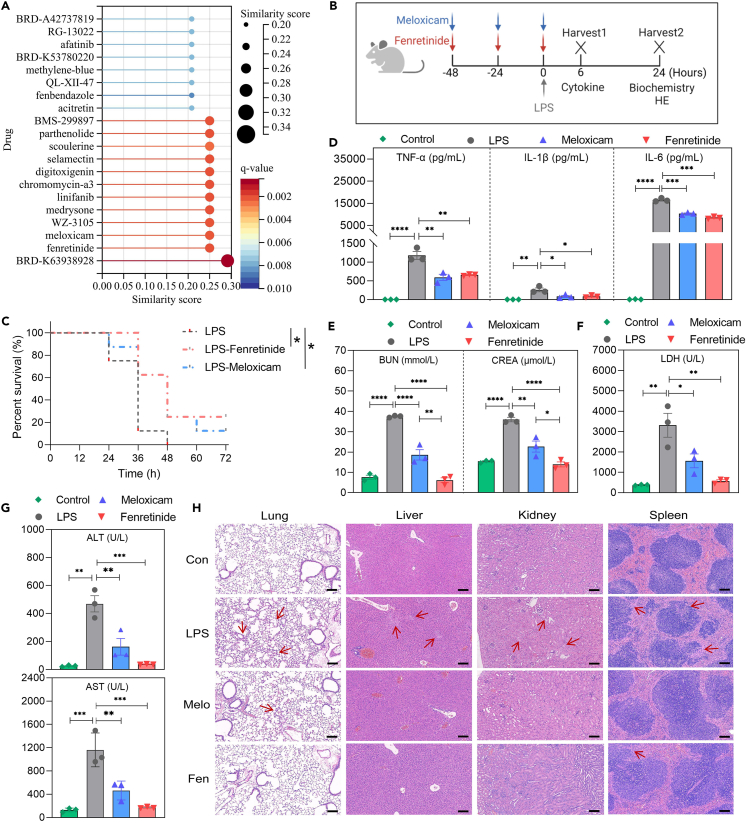
Identified fenretinide (Fen) and meloxicam (Melo) as potential sepsis therapeutics (A) Based on DEGs between HR and LR subgroups, the top 20 potential agents were screened via L1000FWD for sepsis therapy. (B) Schematic of the animal experimental design. Following drug administration for three consecutive days, LPS or PBS (Control) was given by intraperitoneal injection. For survival test, mice were monitored every 12 h till 72 h. For cytokines detection, blood biochemistry, and HE staining, samples were collected after 6 h and 24 h of LPS injection. (C) Survival rates of LPS, Fen-treated, and Melo-treated septic mice were recorded over 72 h. (D) At the 6-h time point, the levels of inflammatory cytokines (TNF-α, IL-1β, and IL-6) in plasma were detected by Elisa tests. (E–G) At the 12-h time point, blood biochemistry tests were performed for (E) kidney (BUN and CREA), (F) heart (LDH), and (G) liver (ALT and AST) function. (H) The HE staining of lung, liver, kidney, and spleen tissues, with a red arrow indicating pathological areas. Magnification: 100×. Size bar: 100 μm. All results were represented as means with error bars representing SEM (*n* = 3). Statistical significance was calculated by ANOVA using Dunnett’s test. ∗*p* < 0.05, ∗∗*p* < 0.01, ∗∗∗*p* < 0.001, ∗∗∗∗*p* < 0.0001.

To explore possible drugs for sepsis treatment, we uploaded 31 common DEGs (Table S5) from risk subgroups (HR vs. LR) across three validation datasets to the L1000FWD, which provided drug prediction targeted to gene expression profiles.^13^ The top 20 significant small-molecule agents were displayed in Figure 6A, with BRD-K63938928, fenretinide (Fen), and meloxicam (Melo) emerging as the most promising candidates for sepsis treatment. Given the unavailability of BRD-K63938928, we tested Fen and Melo’s efficacy in LPS-induced sepsis models. The experimental flow chart for LPS-treated and drug-treated mice were shown in Figure 6B. Fen or Melo were intraperitoneally given at 48 h, 24 h, and 0 h before LPS injection. The Fen and Melo treatments significantly improved the survival rate of mice from 0% to 25% and 12.5%, respectively, compared to the LPS-treated group (*n* = 10 in each group, both *p* < 0.05) (Figure 6C).

Plasma levels of pro-inflammatory cytokines TNF-α, IL-1β, and IL-6, which were significantly elevated after LPS injection, were substantially reduced by Fen and Melo treatment by 0.1–0.7 times, respectively (all *p* < 0.05) (Figure 6D). In addition, both treatments alleviated organ dysfunctions, including the kidney (BUN and CREA), heart (LDH), and liver (AST and ALT) (all *p* < 0.05) (Figures 6E–6G). Histological examination (Figure 6H) demonstrated a reduction in inflammatory cell infiltration and tissue damage in the lungs, liver, kidneys, and spleen in mice treated with Fen or Melo, compared to those with LPS alone. Specifically, fewer edematous alveolar walls in the lung, less vacuolar degeneration in the liver, less dilated renal tubules, and a more precise distinction in the spleen suggested a protective effect against LPS-induced inflammation and organ injury. The collective results indicated that Fen and Melo significantly mitigate LPS-induced inflammatory responses and organ damage in mice, suggesting them as potential therapeutic agents for sepsis treatment in humans.

### Single-cell resolution analysis of the four prognostic genes

**Figure 7:**
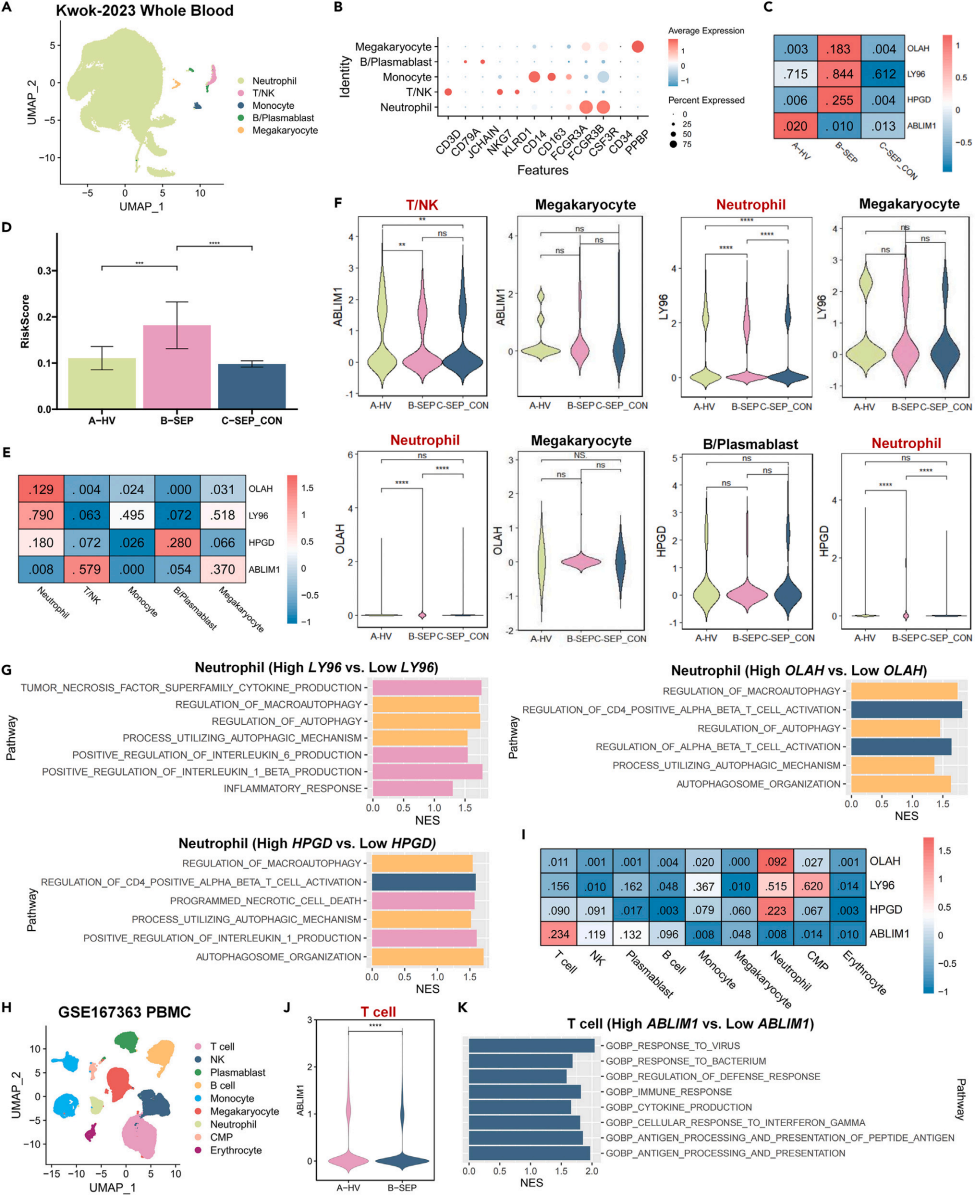
Single-cell analysis of four prognostic genes (A) The UMAP plot of Kwok-2023 scRNA-seq data included whole blood samples from septic patients. (B) The dot plot showed a classical marker expressed in megakaryocyte, B/plasmablast, monocyte, T/NK, and neutrophil clusters. (C) The heatmap indicated overall scaled OLAH, LY96, HPGD, and ABLIM1 expression levels in disease groups (HV: healthy volunteer, SEP: sepsis, SEP_CON: sepsis convalescent). Black values represented the mean expression levels. (D) The risk score in the SEP group was increased compared to HV and SEP_CON groups. (E) The heatmap indicated overall scaled expression levels of OLAH, LY96, HPGD, and ABLIM1 in each cluster. (F) Comparisons of OLAH, LY96, HPGD, and ABLIM1 expression in their respective top two enriched clusters among disease groups. (G) Biological processes enriched in neutrophils with high OLAH, LY96, and HPGD transcripts, as analyzed by GSEA separately. Pathways associated with inflammation responses, T cell activation, and autophagy were colored pink, blue, and yellow, respectively. (H) The UMAP plot of GSE167363 scRNA-seq data included peripheral blood mononuclear cells (PBMC) from septic patients. (I) The heatmap indicated overall scaled expression levels of OLAH, LY96, HPGD, and ABLIM1 in nine clusters in the GSE167363 dataset. (J) The comparison of ABLIM1 expression in T cells among healthy (HV) and sepsis (SEP) groups. (K) Biological processes enriched in T cells with high ABLIM1 transcripts, as analyzed by GSEA. All statistical significance was calculated using the Wilcoxon test. ∗∗*p* < 0.01, ∗∗∗*p* < 0.001, ∗∗∗∗*p* < 0.0001.

Gene functions of the four prognostic genes were further explored in single-cell data of septic patients (Kwok-2023).^14^ After quality control and removal of doublets, five distinct cell types were characterized based on the canonical annotation of marker genes for immune cells, as provided by the original data uploader (Figure 7A and 7B). Megakaryocytes were identified by the expression of PPBP, B/plasma blasts by CD79A and JCHAIN, monocytes by CD14 and CD163, T/natural killer (NK) cells by CD3D, and neutrophils by FCGR3A and FCGR3B. The expression patterns of the four prognostic genes across these cell types were examined, along with their expression levels in the healthy volunteers (HV), septic patients (SEP), and septic-control (SEP-CON) groups. As expected, the risk genes OLAH, LY96, and HPGD were upregulated in the SEP group compared to the HV and SEP-CON groups, while the expression of the protective gene ABLIM1 was decreased (Figure 7C). This resulted in a higher risk score for the SEP group compared to HV (0.18 vs. 0.11, *p* < 0.05) and SEP-CON groups (0.18 vs. 0.10, *p* < 0.05) (Figure 7D).

To identify the specific cell types affected by these signature genes, their mean expression levels across the five cell types were analyzed, and the expression differences within the two most enriched cell types were compared across disease groups. Notably, the genes LY96, OLAH, and HPGD exhibited enriched transcripts and a tendency for upregulation in the neutrophil subset under septic conditions (SEP vs. HV, all *p* < 0.05), highlighting their pivotal roles in neutrophil function (Figure 7E and 7F). Conversely, ABLIM1 showed an enriched transcript and a downregulated trend in the T/NK subset (SEP vs. HV, *p* < 0.05), suggesting its predominant influence on T/NK cells.

A GSEA analysis was further employed to elucidate the underlying intracellular mechanisms of these signature genes within their specific effector cell subsets. By stratifying neutrophils into subclusters based on median expression levels, it was observed that high expression of LY96, OLAH, and HPGD was associated with the regulation of inflammatory responses, autophagy, and T cell activation pathways (Figure 7G). Despite the limited T/NK cell count in the dataset hindering the identification of pathways for ABLIM1, we supplemented our analysis with additional scRNA-seq data from GSE167363, encompassing 46,461 peripheral blood mononuclear cells (PBMCs) from sepsis patients, where T cells and NK cells made up 29.3% and 14.8% of the sample, respectively (Figure 7H). Subsequent analysis confirmed ABLIM1’s highest expression in T cells but not NK cells (Figure 7I). Within T subset, ABLIM1 significantly downregulated in septic patients compared to healthy individuals (SEP vs. HV, *p* < 0.05) (Figure 7J). Importantly, T cells with elevated ABLIM1 expression showed increased activity in biological processes associated with antigen presentation and T cell activation in response to pathogens (Figure 7K). These findings indicated that the risk score encapsulated a broad spectrum of the host’s immune response at the periphery, integrating both innate and adaptive immune responses, and underscored the prognostic genes’ relevance in the sepsis pathophysiology.

## Discussion

In the current study, we constructed a risk score model through a rigorous genomic analysis of differentially expressed genes across independent cohorts. The refined model, comprising genes OLAH, LY96, HPGD, and ABLIM1, demonstrated high predictive accuracy for 28-day mortality and excelled in early sepsis detection. Our analysis revealed the immunosuppressive phenotype of high-risk patients and poor response to steroids in the low-risk subgroup. Drug repositioning has unearthed fenretinide and meloxicam as potential sepsis treatments, enhancing survival in LPS-induced models. Furthermore, single-cell data elucidated the genes’ expression patterns in immune cells, linking them to sepsis pathophysiology. This work highlights the risk score’s clinical utility and the potential of repurposed drugs in sepsis management.

The prognostic genes central to our study have been previously implicated in various biological processes, including innate and adaptive immune responses. LY96, identified as a critical modulator in the innate immune response to LPS via interaction with toll-like receptor (TLR) 4^15^ and TLR2.^16^ In preclinical animal models, competitive binding of LY96 reduced the formation of LPS/TLR4/LY96 complexes, attenuating LPS-induced lung injury and the production of systemic inflammatory cytokines.^17^ HPGD, a critical enzyme in arachidonic acid metabolism, has been linked to organ damage,^18^ bone marrow graft recovery,^19^ and aging.^20^ Inhibition of HPGD in sepsis reduced lipid peroxidation and alleviated LPS-induced kidney injury.^18^ Our finding indicated that neutrophils with highly expressed HPGD and LY96 promote pro-inflammatory responses and autophagy, highlighting their role in inflammatory activation. OLAH, a metabolic enzyme in medium-chain fatty acid biosynthesis,^21^ has been correlated with T cell signaling and poor prognosis in COVID-19 immunopathology, underscoring its potential as a critical factor in T cell activation.^22^ ABLIM1, with its phase-separation properties in actin polymerization control,^23^ was found in high abundance in T cells in our scRNA-seq analysis. The potential role of ABLIM1 in T cell signal transduction through phase separation mechanisms represents an intriguing avenue for future research.

We identified significant downregulation of MHC II-related antigen presentation and T cell activation in the high-risk group, associated with increased 28-day mortalities. MHC molecules are pivotal for adaptive immunity, enabling T cell activation through antigen recognition. Their diminished expression is a hallmark of septic immunosuppression, predisposing patients to secondary infections and heightened mortality risks.^24^^,^^25^^,^^26^ Our results are consistent with Davenport’s work, which characterized the immunosuppressive SRS1 phenotype marked by endotoxin tolerance and T cell exhaustion, linked to higher mortality rates.^8^^,^^27^ Furthermore, our study indicates that the immunologically active low-risk group may suffer adverse outcomes from hydrocortisone treatment, likely due to its inhibitory effect on the highly expressed MHC II molecules in this population.^28^ The differential response to hydrocortisone in our low-risk group adds a layer of complexity to sepsis treatment, suggesting that immunological profiling is crucial for personalized therapeutic strategies.

We also validated two Food and Drug Administration (FDA) approved drugs, Fen and Melo, with potential therapeutic effects in sepsis via animal model validation. Fen, recognized for its anticancer properties, has emerged with anti-inflammatory capabilities, promoting M2 macrophage polarization^29^^,^^30^ and modulating antiviral autophagy pathways.^31^ Its application in acute inflammation models has demonstrated organ-protective effects, rescuing mice from conditions like hepatitis and colitis.^30^^,^^32^ Melo, a COX-2 inhibitor, is an established anti-inflammatory agent for arthritis,^33^ with benefits in sepsis indicated by its modulation of COX-2 upregulation in vital organs and macrophage polarization.^34^^,^^35^ We proved that Fen and Melo significantly alleviated severe inflammation and reduced TNF-α, IL-1β, and IL-6 production in LPS-induced sepsis models, thus warranting further exploration of their role in sepsis clinical treatment.

In conclusion, our study introduced a risk score model with enhanced predictive capabilities for sepsis prognosis and early detection. The identification of potential therapeutic agents through drug repurposing, coupled with insights into immunosuppressive mechanisms, paves the way for fresh treatment strategies that may substantially improve clinical outcomes in sepsis patients.

### Limitations of the study

However, our study has several limitations. First, the diagnostic and prognostic utility of the risk score was assessed primarily using public transcription datasets and a limited number of clinical samples, necessitating large-scale, prospective clinical studies for validation. Secondly, incomplete patient data in public datasets may have limited our ability to account for clinical confounders that could impact the risk score’s accuracy. Additionally, our drug study used a single model of LPS-induced sepsis, thus its generalizability is limited. Future studies adopting the polymicrobial sepsis model induced by cecum ligation and puncture (CLP), which offer a more comprehensive representation of the clinical scenario, will help to validate the broader applicability of our findings. Furthermore, although our findings initially suggested the immunomodulatory mechanisms of the four hub genes and the two drugs, their specific immunomodulatory effects require additional *in vivo* experiments for comprehensive validation.

## Resource availability

### Lead contact

Further information and requests for resources and reagents should be directed to and will be fulfilled by the lead contact, Yali Zheng (YLZheng@xmu.edu.cn).

### Materials availability

This study did not generate new unique reagents.

### Data and code availability

• All RNA sequencing data were available at Gene Expression Omnibus (GEO), ArrayExpress, and European Genome–phenome Archive platforms. Accession numbers are listed in the key resources table.
• This paper does not report original code.
• Any additional information required to reanalyze the data reported in this paper is available from the lead contact upon request.

## Acknowledgments

This work was supported by grants the Science Foundation of Fujian Province (Grant No. 2022J01019) and the 10.13039/100016808Natural Science Foundation of Xiamen City (Grant No. 2022FCX012503010244). We thanked the staff from the Department of Intensive Care Unit in Xiang’an Hospital, Xiamen University, for their assistance in patient recruitment and sample collection.

## Author contributions

Conceptualization, Q.L., Z.G., and Y.Z.; methodology, H.Y. and Q.L.; software, Q.L. and J.L.; validation, S.S., H.Y., and Q.L.; formal analysis, Y.Z., J.W., and Q.L.; investigation, R.L., Q.L., and Z.G.; resources, J.M. and J.L.; data curation, H.Y., S.S., and Q.L.; writing—original draft preparation, Q.L. and H.Y.; writing—review and editing, Q.L. and Y.Z.; visualization, Q.L.; supervision, Z.G. and K.L.; project administration, Y.Z.; funding acquisition, K.L. and J.M. All authors have read and agreed to the published version of the manuscript.

## Declaration of interests

The authors declare no competing interests.

## STAR★Methods

### Key resources table

**Table:** 

REAGENT or RESOURCE	SOURCE	IDENTIFIER
**Biological samples**		

C57/BL6 mice	Xiamen University Laboratory Animal Center	Approval number XMULAC20210021
Human blood samples	Xiangan Hospital of Xiamen University	Approval number XDYX202302K06

**Chemicals, peptides, and recombinant proteins**		

SYBR Green premix reagent	Accurate Biotechnology	Cat#AG11701
Evo M-MLV master mix	Accurate Biotechnology	Cat#AG11706
fenretinide	TargetMol	Cat#T1872
Meloxicam	TargetMol	Cat#T0826
LPS	Sigma-Aldrich	Cat#L2880

**Deposited data**		

GEO data	GEO	GSE167363
EGAS data	European Genome–phenome Archive	EGAS00001006283
ArrayExpress data	ArrayExpress	E-MTAB-5273, E-GEOD-28750, E-MTAB-1548, E-MTAB-4451, E-GEOD-63042, E-GEOD-65682, E-MTAB-7581

**Software and algorithms**		

R v4.2.1	R Core team	www.R-project.org
DESeq2 v1.36.0	Love et al., 2014	https://bioconductor.org/packages/release/bioc/html/DESeq2.html
limma v3.52.4	Matthew et al., 2015	https://bioinf.wehi.edu.au/limma/
Seurat v4.3.0	Satija et al., 2015	https://github.com/satijalab/seurat
glmnet v4.1-8	Friedman et al., 2010	https://glmnet.stanford.edu/
ggrisk v1.3	R CRAN	https://CRAN.R-project.org/package=ggrisk
ggplot2 v3.4.4	H. Wickham. 2016	https://github.com/tidyverse/ggplot2
pROC v1.18.4	Xavier et al., 2011	https://www.expasy.org/resources/proc
rmda v6.7-1	R CRAN	https://CRAN.R-project.org/package=rmda
ClusterProfiler v4.4.4	Wu et al., 2021	https://bioconductor.org/packages/release/bioc/html/clusterProfiler.html
Metascape v3.4	Zhou et al., 2019	https://metascape.org/gp/index.html
GraphPad Prism v8.0	GraphPad	www.graphpad.com

### Experimental model and study participant details

#### Animal experiment

Specific-pathogen-free (SPF) female C57BL/6 mice aged 8–10 weeks were purchased from the Xiamen University Laboratory Animal Center, Xiamen, China. The mice were healthy and were housed in individually ventilated cages for one week’s habituation before experiments. The mouse house was maintained under a standard condition of temperature (23 ± 3°C), relative humidity (55 ± 5%), and a 12-h light/dark cycle. The project has been reviewed and approved by the Ethics Committee of Laboratory Animals of Xiamen University (Permit number: XMULAC20210021).

The L1000FWD tool^13^ was utilized to screen potential sepsis therapeutic drugs. Candidate drugs were identified via similarity query for the core prognostic gene set, defined as the common DEGs among the three validation datasets. We further verified the therapeutic efficacy of candidate drugs in the LPS-induced sepsis mouse models. The candidate drugs fenretinide (30 mg/kg, TargetMol T1872) and meloxicam (30 mg/kg, TargetMol T0826) were at 48h, 24h, and 0h before LPS injection. The mice model used in the survival test was established by intraperitoneal administration of lipopolysaccharide (LPS, Sigma-Aldrich, L2880) at 20 mg/kg.^36^^,^^37^ The mice were monitored every 12 h till 72 h when most of the mice in the LPS group died. Since the high mortality at the dose of 20 mg/kg, we chose a dose of 10 mg/kg for the inflammatory cytokines, biochemistry indicators, and hematoxylin-eosin (HE) assay.^36^^,^^37^ The samples were harvested after 6 h and 24 h after LPS injection.

Under anesthesia, the whole blood of LPS-induced sepsis mice was collected. The blood samples were collected in sterile tubes and centrifuged at 800 g for 10 min after coagulation at room temperature. Then, the plasma was preserved at −80°C for biochemical parameters analysis, and an automatic blood biochemical analyzer was detected. The specific biochemical indicators detected in this study included lactic dehydrogenase (LDH), aspartate aminotransferase (AST), alanine aminotransferase (ALT), blood urea nitrogen (BUN), and creatinine (CREA). Concentrations of commonly proinflammatory cytokines TNF-α, interleukin 6 (IL-6), and interleukin one beta (IL-1β) in plasma were measured through enzyme-linked immunosorbent assay according to instructions of mouse ELISA kits (ABclonal, RK04595, RK04528, and RK04599). The above cytokine concentration was examined at 450 nm absorbance wavelength and calculated with the 4-PL fitting curve.^38^

#### Human subject

The peripheral blood samples were collected from age- and gender-matched (Table S6) patients within 72 h of admission at Xiang’an Hospital of Xiamen University from March 3, 2022, to February 25, 2024. The cohort included 12 sepsis patients, 17 pneumonia patients as disease control, and 6 uninfected pre-operative patients as healthy controls. Sepsis was diagnosed according to the sepsis 3.0 consensus (1). Exclusion criteria included age <18 years old, hospitalization <72 h, hematologic malignant diseases, autoimmune diseases, chronic infectious diseases, and immunosuppressive therapy within the past two weeks. Blood samples were EDTA-anticoagulated and stored at −80°C until analysis.

The study was approved by the ethics committee of the School of Medicine, Xiamen University (Approved number: XDYX202302K06). Before sample collection, consent was obtained from the patients or their guardians.

### Method details

#### Public datasets collection and processing

Six public transcriptome datasets from the Array Express and Gene Expression Omnibus (GEO) database were obtained as discovery and validation cohorts, as detailed in Table S1. Before analysis, the gene expression matrix was log-transformed and normalized. Relative log expression (RLE) and principal component analysis (PCA) were used for quality control.^39^ Next, the “limma” package and “Deseq2” R package were applied to identify differentially expressed genes (DEGs) between the sepsis and control groups for array data and high-throughput transcriptome sequencing (RNA-Seq) data, respectively. DEGs were chosen with an adjusted false discovery rate (FDR) < 0.05 and |log2(FC)| > 1 and displayed via volcano maps using the “ggplot2” package. Common DEGs in the datasets were demonstrated in a Venn diagram.

Two public single-cell transcriptome (scRNA-seq) datasets, Kwok-2023 (EGAS00001006283) and GSE167363, were also included (Table S1). Quality control and cell cluster annotation standards referred to the uploader’s instruction. The “Seurat” package was applied to process the 10× sc-RNA data, including data filtering, normalization, PCA, uniform manifold approximation, and projection (UMAP) analysis. The “dot plot” function generated dot plots illustrating the target genes' relative expression levels in disease groups and cell clusters.

#### LASSO regression and predictive capability validation

The R package “glmnet” was applied for the least absolute shrinkage and selection operator (LASSO) regression analysis, a tool known to exclude inessential variables.^40^ To obtain the optimal model, a 10-fold cross-validation was set. With λ increasing, LASSO tends to reduce the regression coefficient to zero. We chose a λ value of 0.0288 and finally obtained four genes for risk score construction. The “pROC” package obtained the receiver operating characteristic (ROC) curves and area under curves (AUC) values, which were calculated to assess the diagnostic and prognostic capabilities of the risk score. The decision curve analysis (DCA) was generated by the “rmda” package, which estimated a predictive model’s accuracy and net benefit at a range of threshold mortalities based on the medical decision theory.

#### Risk subgroup classification and univariate regression analysis

The validation cohorts were divided into the high-risk (HR) and low-risk (LR) groups by the median risk score. The R package “ggrisk” depicted the risk survival status chart. The survival analysis was performed using the Kaplan-Meier and log rank tests. R packages “rms” and “survival” were applied for univariate Logistic and COX regression analysis. The *p* < 0.05 was considered statistically significant. For risk score construction, DEGs from discovery datasets with |Odds Ratio| > 1 were considered “prognostic genes” and further applied to univariate regression analysis of 28-day mortalities. In validation datasets, risk scores were calculated via LY96, HPGD, OLAH, and ABLIM1 gene expression. Clinical information included 28-day mortality, diabetes, age, pneumonia diagnosis, MARS, and SRS were incorporated into the regression analysis.

#### Functional analysis and correlation analysis

Based on Gene Ontology (GO) and Kyoto Encyclopedia of Genes and Genomes (KEGG) databases, the DEGs from risk subgroups were conducted to function enrichment analysis by Metascape (https://metascape.org/gp/index.html). As for gene set enrichment analysis (GSEA), we used the R package “clusterProfiler” to explore the function associated with risk scores in validation datasets. FDR <0.25 and absolute normalized enrichment score (|NES|) > 1 were statistically significant. The correlation analysis was performed and visualized using the “psych” R package.

#### Clinical sample validation

The RT-PCR experiment validated the OLAH, LY96, HPGD, and ABLIM1 expression levels in clinical samples. Briefly, total RNA was extracted from peripheral blood leukocytes using the TRIzol method, and the relative expression levels were calculated using the 2^−ΔΔCT^ method, with GAPDH as the reference gene. The primer sequences used in this study and qRT-PCR results are shown in Table S2.

### Quantification and statistical analysis

Statistical analyses and visualization of datasets were achieved via R software (Version 4.2.1). For two-group comparisons, the Student’s t test was applied for normal distribution, and the Wilcoxon test was applied for nonnormal distribution with GraphPad Prism software (Version 8.0). The one-way ANOVA analysis with nonparametric Kruskal-Wallis test was applied for multiple-group comparisons in RT-PCR assays of clinical samples. The one-way analysis of Dunnett’s test was applied for multiple-group comparisons in animal experiments. All *p* values were two-sided tests, and *p* < 0.05 was considered statistically significant.
