# Supplementary material for: A transcriptome-based risk model in sepsis enables prognostic prediction and drug repositioning
Source: iScience. 2024 Oct 28;27(12):111277. doi: 10.1016/j.isci.2024.111277 (PMC11613189; doi:10.1016/j.isci.2024.111277)
Supplement: Document S1. Figures S1–S3 and Tables S1–S6 [file mmc1.pdf]

iScience, Volume *27*

Supplemental information

A transcriptome-based risk model in sepsis enables prognostic prediction and drug repositioning

Qiuyue Long, Hongli Ye, Shixu Song, Jiwei Li, Jing Wu, Jingsong Mao, Ran Li, Ke Li, Zhancheng Gao, and Yali Zheng

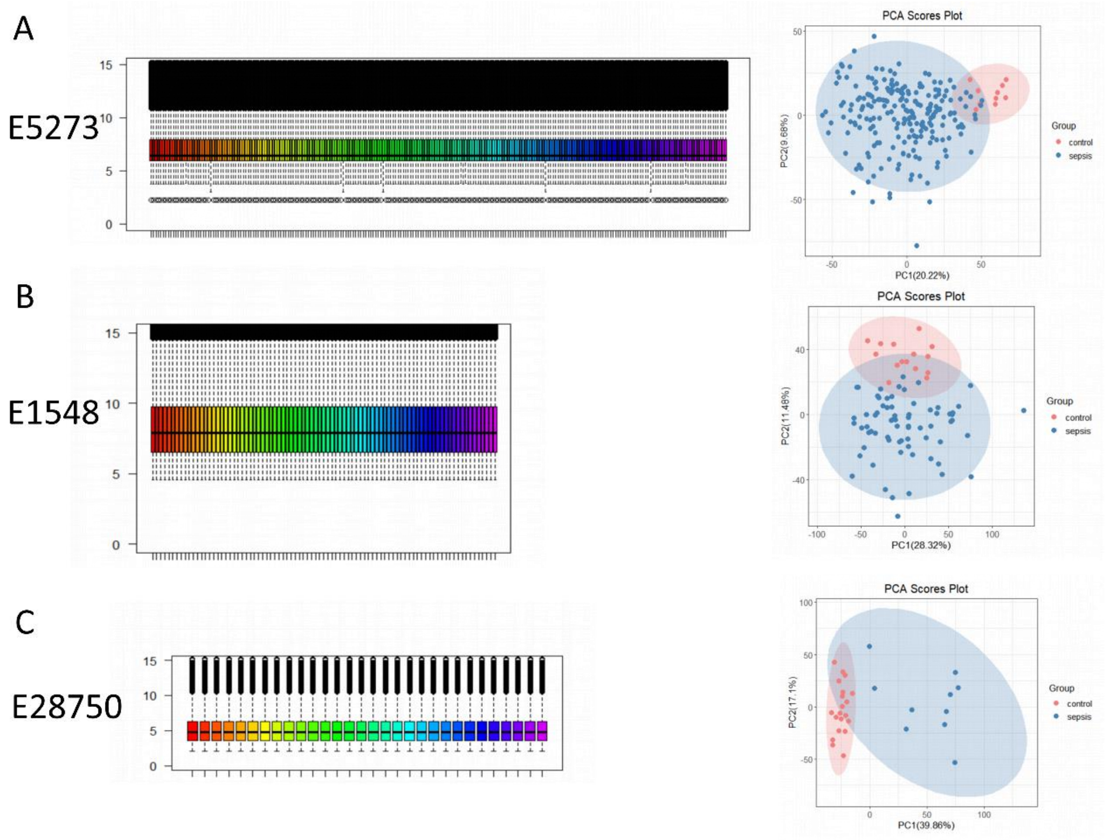

Figure S1. Data preprocessing for quality control. The RLE visualization and PCA analysis after processes of log-transformation and normalization in the dataset (A) E5273, (B) E1548, and (C) E28750.

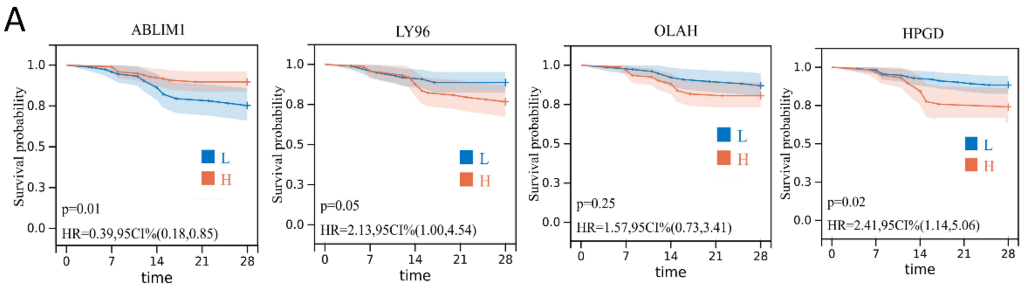

Figure **S2.** The Survival analysis of four prognostic genes. (A) The Kaplan-Meier curves of four prognostic genes. L: Low-expression group, H: High-expression group.

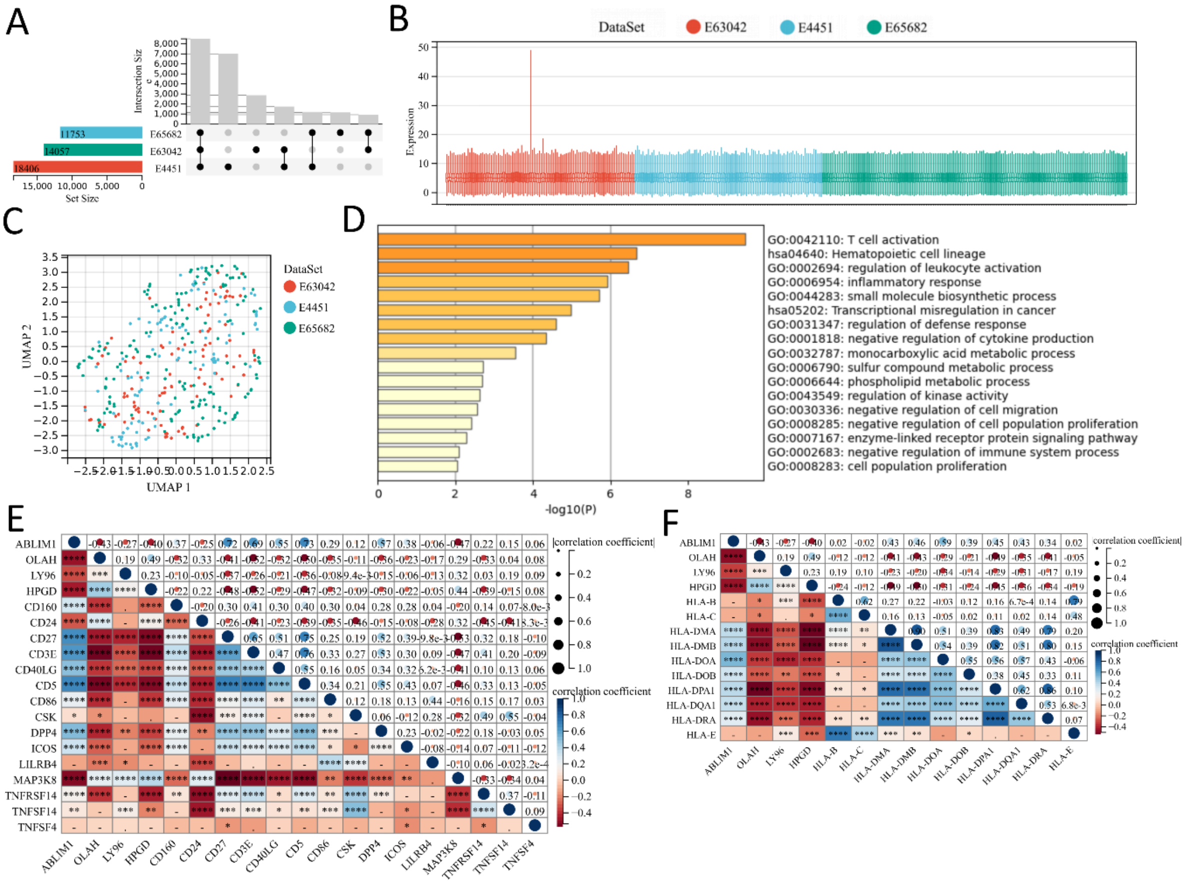

Figure S3. Function analysis of risk subgroups in the combined validation dataset. (A) The Venn graph showed shared 8, 497 genes in three validation datasets. After combining three datasets and removing batch effect, (B) the boxplot indicated consistent trends of data distribution in all datasets after removing batch effect, and (C) the UMAP plot showed that each dataset was interwoven. (D) The GO and KEGG enrichment analysis of DEGs generated from riskscore groups in the combined dataset. The closer the color is to deep, the smaller the *P* is. Spearman correlation analysis of 4 signature genes and (E) co-stimulators or (F) major histocompatibility complex (MHC) molecules.

Table S1. Characteristics of transcriptomic datasets included in this study.

	Dataset	Experiment type	Sample	Group (n)			
				Healthy control	SIRS	Sepsis	Sepsis convalescent
**Discovery sets**	E-MTAB-5273	Array	whole-blood leukocyte	10	-	221	-
	E-GEOD-28750	Array	whole-blood leukocyte	20	-	10	-
	E-MTAB-1548	Array	whole-blood leukocyte	15	-	70	-
**Validation sets**	E-MTAB-4451	Array	whole-blood leukocyte	-	-	106	-
	E-GEOD-63042	RNA-Seq	whole-blood leukocyte	-	-	106	-
	E-GEOD-65682	Array	whole-blood leukocyte	-	-	183*	-
**Drug responses set**	E- MTAB-7581	Array	whole-blood leukocyte	-	-	176	-
**scRNA-seq sets**	EGAS00001006283	scRNA-seq	whole-blood leukocyte	6	-	26	9
	GSE167363	scRNA-seq	Peripheral blood mononuclear cells	1	-	5	-

* In E-GEOD-65682 dataset, the 183 patients met diagnostic criteria of sepsis-3 definition were included sepsis cohort. The criteria included infectious diagnosis (106 community-acquired pneumonia and 77 hospital-acquired pneumonia) and sequential organ failure assessment (SOFA) scores of 2 points or more.

Table S2. Primers of human species used in this study.

	Primers
**h-OLAH-F**	GGAGACCAACCTAAGAGAACCA
**h-OLAH-R**	AGCAAATCAGCTTAAAAGTTGCC
**h-ABLIM1-F**	TGAGCCTTTCTACACTTCGGG
**h-ABLIM1-R**	TCTGCTGATGGAGTAGGAGAC
**h-HPGD-F**	GCTGGTGGATTGGAATCTTGA
**h-HPGD-R**	TCTCTCAGTTGTTGCTGGTCA
**h-LY96-F**	GAAGCAGTATTGGGTCTGCAA
**h-LY96-R**	TTGGAAGATTCATGGTGTTGACA
**h-GAPDH-F**	GAAATCCCATCACCATCTTCC
**h-GAPDH-R**	GAGCCCCAGCCTTCTCCATG

Table S3. The 20 significant genes relevant to 28-day death event by univariate cox regression analysis.

Gene symbol	Gene name	log2FC in E5273 dataset	log2FC in E1548 dataset	log2FC in E28750 dataset	Hazard Ratio (95%CI)	P value
**OLAH**	Oleoyl-ACP Hydrolase	2.615308655	1.629356513	1.705563008	1.4 (1.2-1.8)	< 0.01
**HPGD**	15-Hydroxyprostaglandin Dehydrogenase	1.788894357	1.126319588	2.621822864	1.4 (1.2-1.7)	< 0.01
**ABLIM1**	Actin Binding LIM Protein 1	-1.321704005	-1.213004365	-2.276897879	0.57 (0.39-0.84)	< 0.01
**ST6GALNAC3**	ST6 N-Acetylgalactosaminide Alpha-2,6-Sialyltransferase 3	1.490116805	1.017119719	2.16411993	1.6 (1.1-2.4)	0.01
**TLR5**	Toll Like Receptor 5	2.334942659	1.012725984	2.33077332	2 (1.2-3.3)	0.011
**TGFBI**	Transforming Growth Factor Beta Induced	-1.694864943	-1.06231911	-1.720260961	0.75 (0.59-0.94)	0.012
**UPP1**	Uridine Phosphorylase 1	2.052759601	1.055472257	1.962632085	1.7 (1.1-2.7)	0.019
**TPST1**	Tyrosylprotein Sulfotransferase 1	1.665259653	1.035321264	1.086261005	1.5 (1.1-2.1)	0.02
**CCR3**	C-C Motif Chemokine Receptor 3	-2.746797395	-1.114913705	-2.68261846	0.78 (0.62-0.97)	0.026
**DHRS9**	Dehydrogenase/Reductase 9	1.684359911	1.178384942	1.744672821	1.4 (1-1.9)	0.026
**IL1R2**	Interleukin 1 Receptor Type 2	2.266422241	1.755470274	3.150920743	1.4 (1-1.9)	0.027
**ARG1**	Arginase 1	3.650201931	1.912069208	4.762207329	1.3(1-1.7)	0.029
**LY96**	Lymphocyte Antigen 96	1.544658664	1.033149141	2.207881854	1.9(1.1-3.3)	0.029
**IL7R**	Interleukin 7 Receptor	-2.021614274	-1.357199237	-2.373751495	0.74(0.56-0.97)	0.031
**GRB10**	Growth Factor Receptor Bound Protein 10	2.301512878	1.248580498	3.163343724	1.5(1-2.1)	0.032
**HLA-DRA**	Major Histocompatibility Complex, Class II, DR Alpha	-2.018093446	-1.006027592	-2.147383352	0.75(0.58-0.98)	0.032
**IRAK3**	Interleukin 1 Receptor Associated Kinase 3	2.294340001	1.119985622	3.211322717	1.6(1-2.4)	0.034
**DAAM2**	Dishevelled Associated Activator Of Morphogenesis 2	1.842999315	1.255105292	1.585891991	1.3(1-1.6)	0.041
**IL2RB**	Interleukin 2 Receptor Subunit Beta	-2.489286798	-1.155368008	-2.366837896	0.71(0.51-0.99)	0.043
**LEF1**	Lymphoid Enhancer Binding Factor 1	-1.924720744	-1.537252038	-2.654550333	0.76(0.58-0.99)	0.044

Table S4. RT-PCR results of clinical samples and risk scores calculated by the relative expression levels of genes HPGD, LY96, OLAH, and ABLIM1.

Group	HPGD	ABLIM	LY96	OLAH	Risk score
**Pre-op 1**	2.788187668	2.465624058	1.234800888	0.202547846	0.290705491
**Pre-op 2**	1.814230108	1.925836688	2.775267118	0.628762126	0.556814643
**Pre-op 3**	0.758567223	0.915321863	0.803251106	0.635822802	0.222522063
**Pre-op 4**	0.612882537	0.595663724	0.411377052	0.174763891	0.101793664
**Pre-op 5**	0.731550275	0.488847967	0.542550096	1.000013941	0.260609776
**Pre-op 6**	1.24620907	0.901502568	0.834977669	0.249331136	0.21223873
**Pneumonia 1**	1.0000649	1.000505578	1.000019878	0.07561952	0.186328985
**Pneumonia 2**	0.290229822	0.237028024	1.688060233	0.058568453	0.287612385
**Pneumonia 3**	0.0939379	3.195268556	0.278933175	0.095157053	-0.150246452
**Pneumonia 4**	2.452193674	0.168743161	1.685227925	1.298909572	0.651514037
**Pneumonia 5**	0.093391382	0.077330027	2.386557975	0.228911549	0.419341122
**Pneumonia 6**	1.491846312	0.509011672	0.14937941	0.214154713	0.14383084
**Pneumonia7**	3.992833793	0.639876366	1.000459007	1.003114161	0.593340205
**Pneumonia8**	1.002260431	1.001096062	0.773304074	0.720502372	0.244377002
**Pneumonia9**	2.333794235	3.476428784	0.698332451	0.218405865	0.100808593
**Pneumonia10**	0.138689129	0.958787415	0.698332451	0.753085796	0.168749922
**Pneumonia11**	0.221861316	1.011482277	0.110919497	0.109060643	-0.01653632
**Pneumonia12**	3.286333065	2.617820943	1.39846038	0.659402127	0.414581839
**Pneumonia13**	0.12289514	2.035805507	1.544198048	0.081651278	0.132072955
**Pneumonia14**	1.100763	1.048123	1.239149	1.057671	0.373467379
**Pneumonia15**	2.194992	1.687694	0.416676	0.449654	0.199261881
**Pneumonia16**	0.488132	1.427234	1.239149	0.2482	0.178826436
**Pneumonia17**	1.042654	0.560491	0.829264	0.165075	0.20533888
**Sepsis 1**	3.079664254	0.101744392	2.01827408	52.70853072	8.281145223
**Sepsis 2**	0.51166575	0.256661202	0.678431344	22.1049072	3.36712807
**Sepsis 3**	1.998170541	0.059671867	0.311029878	11.62371843	1.910943752
**Sepsis 4**	57.4549221	0.056282519	2.055697502	156.262099	27.92164342
**Sepsis 5**	6.812930981	0.123467834	12.35961145	128.7965848	21.37851202
**Sepsis 6**	4.964921535	0.041291066	0.424449424	36.41670381	5.801621294
**Sepsis 7**	6.776876426	0.36999631	6.695505233	28.69535131	5.806778546
**Sepsis 8**	5.133763	0.183488	3.468971	15.00967	3.163733611
**Sepsis 9**	6.54	0.165392	1.805534	41.90543	6.947824064
**Sepsis 10**	2.544653	0.211648	1.754825	10.32492	1.987670748
**Sepsis 11**	1.741193	0.286141	2.640542	1.687694	0.795255149
**Sepsis 12**	3.041113	0.203291	1.603849	7.254131	1.555753119

Table S5. Common DEGs generated from LR and HR subgroups in three validation datasets for drug screening.

E4451/GSE65682/E63042		E4451/GSE65682	
**Gene**	**Expression status**	**Gene**	**Expression status**
OLAH	Up	CD3D	Down
GRB10	Up	CCR7	Down
ASPH	Up	IL7R	Down
IL1R2	Up	LEF1	Down
HPGD	Up	GNLY	Down
IL18R1	Up	GZMH	Down
ST6GALNAC3	Up	SULF2	Down
DAAM2	Up		
KCNE1	Up		
PFKFB2	Up		
ADORA3	Up		
TDRD9	Up		
ARG1	Up		
IDI1	Up		
CLEC4D	Up		
KCNMA1	Up		
CYP19A1	Up		
ZDHHC19	Up		
CD177	Up		
FGF13	Up		
SMPDL3A	Up		
ANKRD34B	Up		
MMP8	Up		

Table S6. The basic information of included patients in this study.

Characteristics	Pre-operative (n = 6)	Pneumonia (n =17)	Sepsis (n = 12)	p value
**Age, median (IQR)**	61 (41, 67)	57 (40, 62)	63 (54, 71)	0.490
**Sex, n (%)**				0.092
Male	0 (0.00%)	7 (41.18%)	6 (50.00%)	
